# Satisfaction in Patients After Rhinoplasty Using the Rhinoplasty Outcome Evaluation Questionnaire

**DOI:** 10.7759/cureus.5283

**Published:** 2019-07-30

**Authors:** Nasir Khan, Mamoon Rashid, Ibrahim Khan, Saad Ur Rehman Sarwar, Haroon Ur Rashid, Mariam Khurshid, Usama Khalid Choudry, Noor Fatima

**Affiliations:** 1 Plastic Surgery, Shifa International Hospital, Islamabad, PAK; 2 Dermatology, Pakistan Air Force Hospital, Islamabad, PAK; 3 General Surgery, Shifa International Hospital, Islamabad, PAK; 4 Internal Medicine, Khyber Girls Medical College, Peshawar, PAK

**Keywords:** rhinoplasty, satisfaction, roe questionnaire

## Abstract

Introduction

Rhinoplasty is a challenging procedure. The goal of the surgery is not only to restore the function and youthful appearance of the nose but also to improve quality of life. With the passage of time, the trend has been changing rapidly from more invasive to less invasive procedures. Although the technical aspects of rhinoplasty are important, patient satisfaction is the factor that dictates the success of the procedure.

Materials and methods

A total of 118 rhinoplasties were performed in our department between 2016 and 2018. The Rhinoplasty Outcome Evaluation (ROE) questionnaire was used to study the patients' satisfaction level. Ninety out of 118 patients took part in this study. Rhinoplasty was done using an open technique in all cases. The ROE questionnaire was filled preoperation and six months postoperation. Data analysis was done using SSPS statistic version 20 (IBM Corp., Armonk, NY, US).

Results

The main reasons for rhinoplasty in our patients were: aesthetic 23.3% (n=21), functional 25.5% (n=23), and a combination of both in 51% (n=46) patients. The mean ROE score of all patients preoperation was 30.5 (males: 31.3, females 29.8) and the mean score postoperation was 79.5 (males 78.2, females 80.9) at six months with no statistical differences (CI 17.11 - 12.59, P=0.762). However, both genders showed a statistically significant improvement between the preoperative and postoperative scores (mean difference = 49.3, CI 63.25 - 35.34, P<0.01), indicating an overall good satisfaction level after surgery. The satisfaction level of patients was inversely proportional to their level of understanding and knowledge of the surgical procedure. This difference was statistically significant ( CI 7.36-10.42, P<0.01). Minor corrections or modifications were done in eight patients under local anesthesia, with no significant difference in ROE scores as compared to those who had single surgery (CI 0.7 - 1.6, P=0.92). There was no statistically significant difference in the before and after surgery ROE scores among patients operated by different surgeons as well (P=0.82).

Conclusion

Our study shows that rhinoplasty, despite being a complex procedure, has proven benefits in terms of functional as well as aesthetic outcomes. The ROE questionnaire proves to be a valid tool for estimating patient satisfaction in our population. There is a need for further training and education of surgeons in Pakistan to improve the functional and aesthetic disabilities of nasal deformities.

## Introduction

Rhinoplasty is one of the most commonly performed plastic surgery procedures for both functional and aesthetic purposes according to the statistics of the American Society of Plastic Surgeons [1]. In Asia, the demand for aesthetic rhinoplasty has significantly increased in the last two decades due to increasing self-attention, media awareness, and advancement in surgical manoeuvers. The trend has been seen mostly in the young age group among both men and women [2].

This procedure is considered one of the most technically demanding of all plastic surgery procedures. The surgeon must understand the underlying anatomy, have the ability to perform a nasofacial analysis to determine the operative plan, and execute techniques that manipulate bone, cartilage, and soft tissue. These techniques are augmented by an aesthetic eye in order to produce a result that blends harmoniously with the rest of the face [3]. One of the main goals of surgery is to improve the patient’s appearance and ultimately relieve his or her social anxiety and persecution complex [4].

The patient’s satisfaction varies based on gender, age, education level, culture, ethnicity, and, most importantly, the patient’s level of expectation [5]. Assessing factors contributing to the patient’s satisfaction is the main focus in preoperative evaluation. Due to the diversity of the procedure and the difficulty in interpreting patient expectations, the post-rhinoplasty satisfaction rate is low [6]. Patient selection is very crucial in rhinoplasty, as a significant percentage of patients may not be satisfied despite a good surgical result [7].

There are multiple patient-reported outcome measures available to evaluate pre and postoperative patient satisfaction and quality of life in patients undergoing rhinoplasty. These tools are categorized into three groups: measuring the outcomes of aesthetic, functional, and combined [8]. The facial appearance sorting test (FAST) can be used for the assessment of the rhinoplasty outcome. The Derriford Ford Appearance Scale (DAS-59) can be used to assess the effect of appearance on quality of life [9].

The Rhinoplasty satisfaction outcome evaluation is a tedious task to perform, especially when it is being performed by different consultants. To overcome this difficulty, we use the Rhinoplasty outcome evaluation (ROE) questionnaire to access our results [10]. ROE is a quick and easy-to-perform questionnaire, which is a standardized and reliable method of evaluating quality of life following rhinoplasty. It measures qualitative aspects such as social, emotional and psychological variables [11]. Due to increasing demand and levels of expectations among patients, we conducted a study to assess the satisfaction level of our patients before and after the surgery.

## Materials and methods

After approval from the ethical committee, a prospective observational study of 90 patients was performed. Rhinoplasties performed from January 2016 till December 2018 were evaluated. Patients from both genders, between 16 and 60 years of age, were included in the study. Patients with congenital nasal deformities, such as cleft nasal deformities, craniofacial clefts, and those requiring secondary rhinoplasties, were excluded from the study. Informed consent was taken. Before and after surgery, digital photography was done in all patients for recordkeeping, planning, and comparison. Preoperative patient’s concerns were documented, and detailed counseling was done. The ROE questionnaire was discussed and explained to all the patients. The rhinoplasty outcome evaluation scale consists of six questions that study three quality of life parameters, i.e. physical, emotional, and social. The open rhinoplasty approach was done in all patients with the use of a septal cartilage graft in 69 patients, conchal cartilage graft in 12 patients, and rib cartilage in nine patients. The questionnaire was filled preoperatively and at six months' follow-up in all patients. All the patients' data were compiled and outcomes were assessed.

Data were recorded in terms of mean ± S.D. Measures of central tendency and variance were calculated. The student's *t*-test was applied for statistical correlation. The confidence interval was kept at 95%. P<0.05 was considered statistically significant. Data analysis was done using SPSS 20.0.1 (IBM Corp., Armonk, NY, US).

## Results

Out of 118 patients, 90 answered the questionnaires and were included in the study. Around 64.4% (n=58) were females and 35.5% (n=32) were male. The mean age was 22.5±2.6 years in females and 28.2±2.8 years in males. The indications for rhinoplasty in patients were aesthetic 23.3% (n=21), functional 25.5% (n=23), and a combination 51% (n=46) of both in our study population. The mean ROE score of all patients preoperation was 30.5 (males 31.3, females 29.8) and the mean score post-operation was 79.5 (males 78.2, females 80.9) at six months. All patients showed a statistically significant improvement between the preoperative and postoperative scores (mean difference = 49.3, CI 63.25 - 35.34, P<0.01), indicating a good satisfaction level after surgery, however, There was no statistical difference in ROE score improvement, when comparing both sexes (CI 17.11 - 12.59, P=0.762). The mean scores of patients on an individual's questions of ROE are depicted in Table 1.

**Table 1:** Mean scores of patients in the ROE questionnaire on the Likert scale ROE: Rhinoplasty outcome evaluation

Questions	Mean Pre-operative Score	Mean Post-operative score
How much do you like the appearance of your nose?	1.2±0.5	3.1±0.4
How much can you breathe through your nose?	1.4±0.6	3.2±0.5
How much do you think your friends and close ones like your nose?	1.0±0.5	2.8±0.7
Do you think your current nasal appearance limits your social or professional activities?	1.3±0.7	3.0±0.8
How confident are you that your nasal appearance is the best it can be?	1.2±0.8	3.3±0.6
Would you like to surgically alter the appearance or function of your nose?	1.1±0.4	3.6±0.5
Total score	7.2/24	19/24

**Figure 1:**
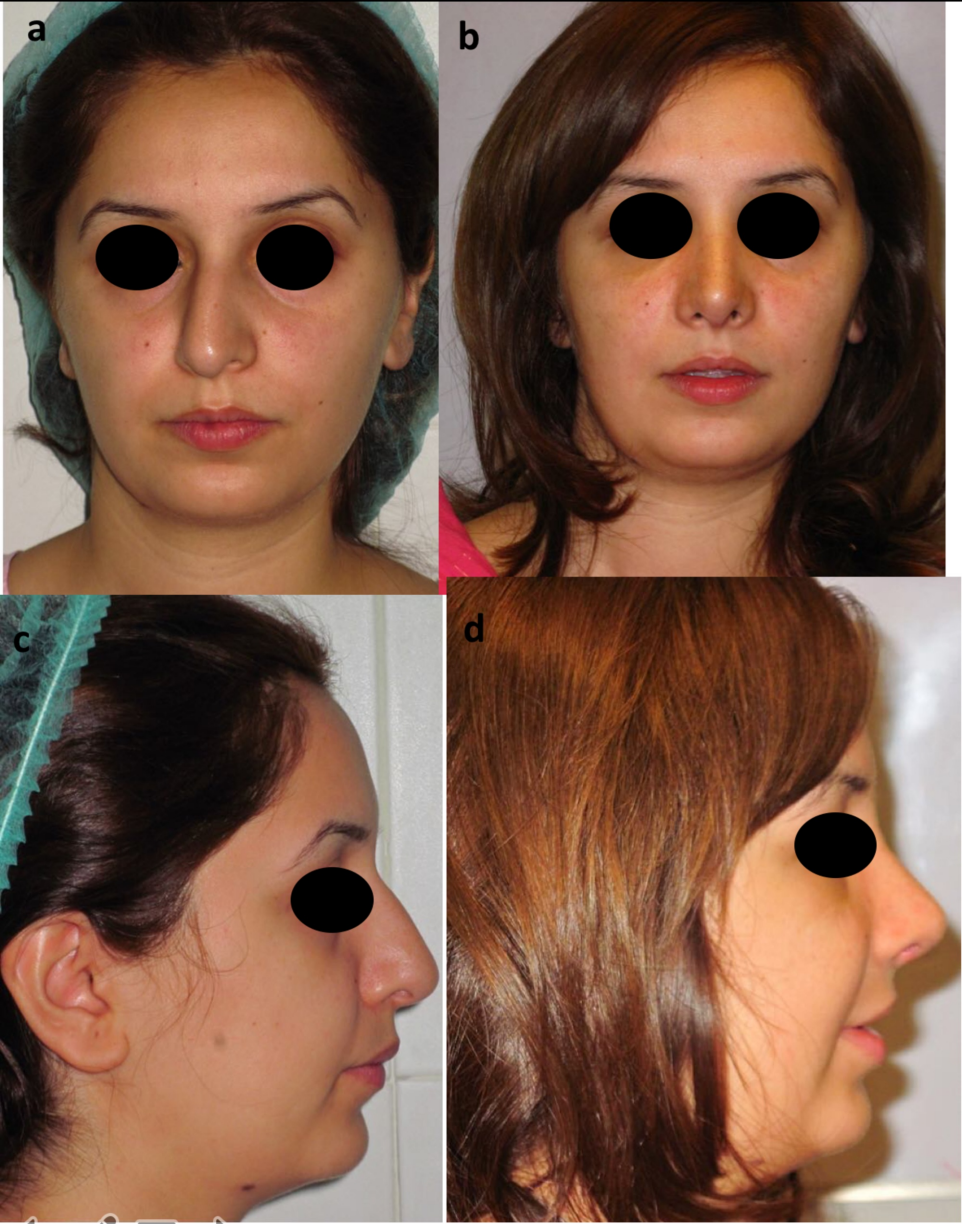
Frontal and lateral views Frontal view photographs are shown before (above, left a) and six months after rhinoplasty, dorsal hump reduction, correction of septal deviation, columellar strut, lateral crural strut, interdomal suturing, and spreader graft placement (above, right b). Lateral view photographs before (below, left c) and after (below, right d) surgery demonstrate a smooth dorsum, good tip elevation, and appropriate nasolabial angle.

**Figure 2:**
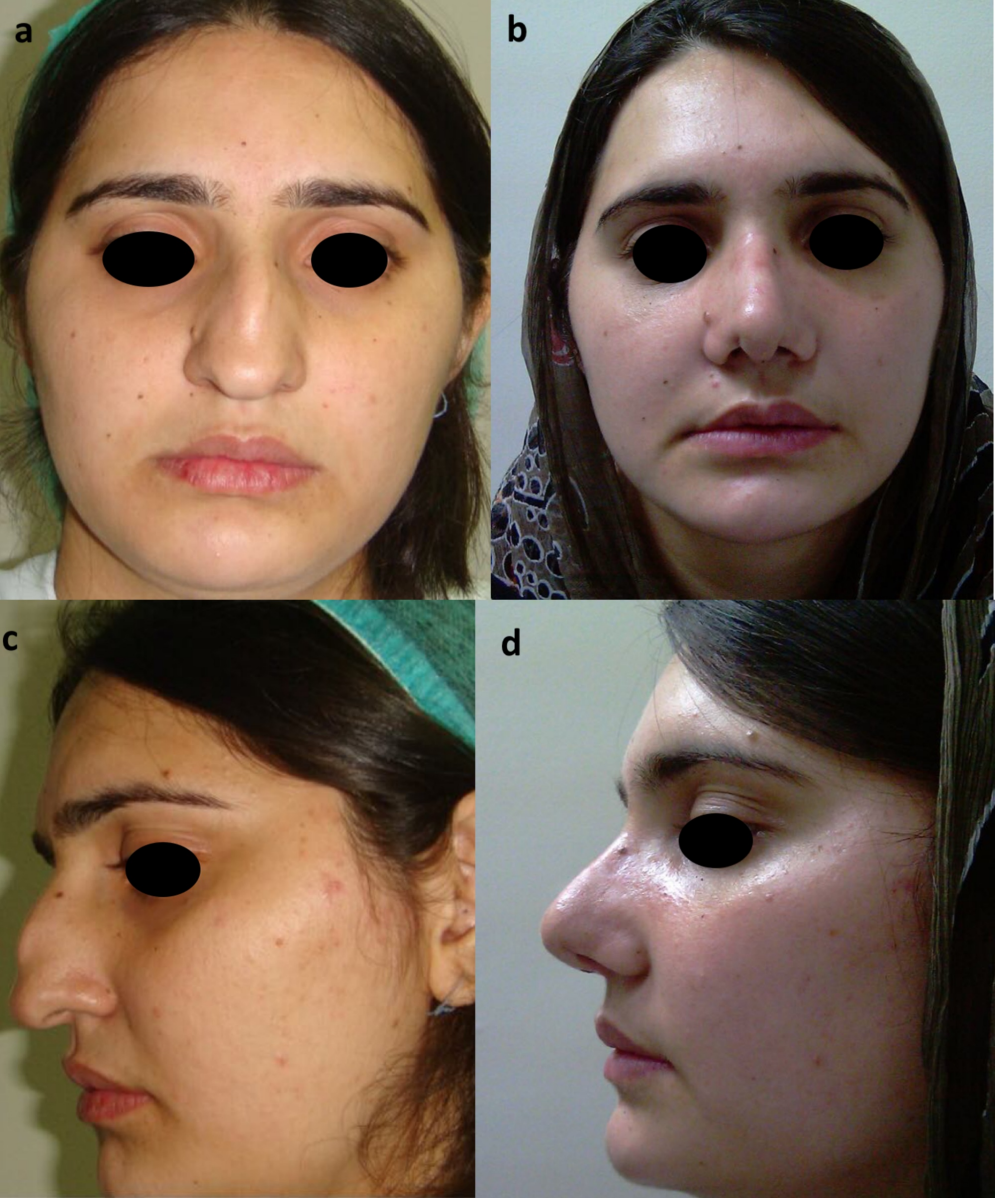
Frontal and lateral views Frontal view photographs are shown before (above, left a) and six months after rhinoplasty (above, right b). Lateral view photographs before (below, left c) and after (below, right d).

The satisfaction level of patients was inversely proportional to their level of understanding and knowledge of the surgical procedure. In our population, about 28 patients had a low literacy level (ninth grade and below ) while 52 had a higher literacy level (postgraduate and above). The mean postoperation score in patients with a low literacy level was 81.4±4.6 as compared to 72.5±2.7 in individuals with a high literacy level. This difference was statistically significant (CI 7.36-10.42, P<0.01). Minor corrections or modifications were done in eight patients under local anesthesia, with no significant difference in ROE scores as compared to those who had single surgery (CI 0.7 - 1.6, P=0.92). There was no statistically significant difference in the preoperation and postoperation ROE scores among patients operated by different surgeons as well (P=0.82). The surgical approach was open rhinoplasty in all cases, with the use of a septal cartilage graft in 70 (77%), conchal cartilage graft in 12 (13%), and rib cartilage graft in 8 (8.8%) patients. There was no statistical difference in patient satisfaction with regards to the type of graft used (P=0.54). Septoplasty combined with rhinoplasty was done in 53 patients. Overall, our patients had improved post-surgery ROE scores, which shows that maximum satisfaction was achieved. There were no major complications except for minor wound infections in 4 (4.4%) patients, which were managed conservatively. There was no donor site morbidity or deformity seen in any patient. In 8.8% (n=8) of our patients, minor corrections were done in the follow-up period i.e. tip plasty (n=5) and Weir resections (n=3), which were done under local anesthesia as daycare procedures. Figures 1-2 show the frontal and lateral views of some patients.

## Discussion

Rhinoplasty is a challenging procedure. The goal of the surgery is not only to restore the function and youthful appearance of the nose but also to improve quality of life. With the passage of time, the trend has been changing rapidly from more invasive to less invasive procedures. Although the technical aspects of the rhinoplasty are important, patient satisfaction is the factor that dictates the success of the procedure [12]. The measurement of patient satisfaction is a difficult task, with no real standards available. JM Herruer et al. studied the influence of psychological aspects like self-consciousness of appearance and expectations from surgery [13]. They postulated that patients seeking surgery are distressed due to self-consciousness of appearance [14]. There may be unexpected responses from patients even after good surgical corrections because rhinoplasty has a huge psychological impact [15].

According to another study conducted among patients with psychological abnormalities, 80% were satisfied with the surgical outcomes [16]. As compared to other aesthetic procedures, rhinoplasty patients are less satisfied with their appearance after surgery [17].

In 2000, Alsarraf et al. were the first to create a questionnaire, which is reliable for several plastic surgery procedures [18-19]. It was later modified by Arima et al. [20] for those having rhinoplasty, and it was called the rhinoplasty outcome evaluation (ROE) questionnaire. The ROE scale consists of six questions that study three qualitative aspects: the physical, psychological, and social aspects. A postoperative score above 80% is considered excellent, and it means that the patient is very satisfied [21]. A recent study suggested that meeting aesthetic expectations was more important than meeting functional expectations to satisfy a patient [22]. A gain of a minimum of 36 in the ROE scale is considered an improvement [12].

In our study, individuals with low literacy levels showed psychological stability with lower expectations from the surgery. They also needed fewer counseling sessions. On the other hand, patients with more information and knowledge of the procedure had higher expectations from the surgery, requiring multiple sessions to improve understanding with the surgeon [11].

In our study, the mean ROE preoperation score was 30.5 and the mean postoperation score was 79.5 at six months, with a mean gain of 49 points after surgery. These numbers are comparable to those reported by Alsaraf et al. [19], in which the mean preoperation score was 38.8 and the mean postoperation score was 83.3, with a mean gain of 44.5. Sena Esteves et al. [11] also showed similar results with a mean preoperation score of 32.78 and a mean postoperation score of 81.9 with a mean gain or improvement of 49.3.

Our study showed a slightly higher satisfaction score in females as compared to males regarding the procedure (females 80.9, males 78.2). These findings are in agreement with the study conducted by Khansa et al., reporting higher satisfaction in females [6]. Another review of male rhinoplasties states that male patients usually have nonspecific complaints and have a poor understanding of their deformity [23]. A detailed comparison of previously reported literature with our results is shown in Table 2.

**Table 2:** Comparison of rhinoplasty outcomes evaluation scores with previously reported literature

Reference	Study design	Preoperatively	Postoperatively	Change
Meningaud et al., 2008 [21]	Prospective	40.66	70.7	30.4
Arima et al., 2012 [24]	Prospective	27.2±10.8	77.7±17.2	50.5
Cingi and Eskiizmir, 2013 [25]	Prospective	19.77±7.99	76.20±17.46	56.32
Günel and Omurlu, 2015 [26]	Prospective	45(range 75-90)	80(range 75-90)	35
Bulut et al., 2015 [27]	Prospective	42.2±15.7	63.9±18.9	21.7
Present Study	Prospective	30.5	79.5	49

Our study shows preliminary data regarding patient satisfaction based on a single evaluation tool. There is a need for a study with a larger sample size and more specific quality-of-life tools to further validate the benefits of rhinoplasty.

## Conclusions

Our study shows that rhinoplasty, despite being a complex procedure, has proven benefits in terms of functional as well as aesthetic outcomes. The rhinoplasty outcome evaluation questionnaire proves to be a valid tool for estimating patient satisfaction in our population. It can be concluded from our study that rhinoplasty provides long-term satisfaction in the majority of patients. There is a need to further train and educate surgeons in this technique to improve the outcomes of the functional and aesthetic disabilities of nasal deformities in Pakistan.
